# Emergency Telemedicine Mobile Ultrasounds Using a 5G-Enabled Application: Development and Usability Study

**DOI:** 10.2196/36824

**Published:** 2022-05-26

**Authors:** Maximilian Berlet, Thomas Vogel, Mohamed Gharba, Joseph Eichinger, Egon Schulz, Helmut Friess, Dirk Wilhelm, Daniel Ostler, Michael Kranzfelder

**Affiliations:** 1 Department of Surgery Klinikum Rechts der Isar Technical University Munich Munich Germany; 2 German Research Center, Munich Office Huawei Technologies Düsseldorf GmbH Munich Germany; 3 Researchgroup Minimally-Invasive Therapy and Intervention Klinikum Rechts der Isar Technical University Munich Munich Germany

**Keywords:** 5G, telemedicine, telehealth, eHealth, digital health, digital medicine, mobile ultrasound, ultrasound, imaging, digitalized medicine, emergency care, emergency, ambulance, slicing, diagnostic, diagnosis, image quality, field test

## Abstract

**Background:**

Digitalization affects almost every aspect of modern daily life, including a growing number of health care services along with telemedicine applications. Fifth-generation (5G) mobile communication technology has the potential to meet the requirements for this digitalized future with high bandwidths (10 GB/s), low latency (<1 ms), and high quality of service, enabling wireless real-time data transmission in telemedical emergency health care applications.

**Objective:**

The aim of this study is the development and clinical evaluation of a 5G usability test framework enabling preclinical diagnostics with mobile ultrasound using 5G network technology.

**Methods:**

A bidirectional audio-video data transmission between the ambulance car and hospital was established, combining both 5G-radio and -core network parts. Besides technical performance evaluations, a medical assessment of transferred ultrasound image quality and transmission latency was examined.

**Results:**

Telemedical and clinical application properties of the ultrasound probe were rated 1 (very good) to 2 (good; on a 6 -point Likert scale rated by 20 survey participants). The 5G field test revealed an average end-to-end round trip latency of 10 milliseconds. The measured average throughput for the ultrasound image traffic was 4 Mbps and for the video stream 12 Mbps. Traffic saturation revealed a lower video quality and a slower video stream. Without core slicing, the throughput for the video application was reduced to 8 Mbps. The deployment of core network slicing facilitated quality and latency recovery.

**Conclusions:**

Bidirectional data transmission between ambulance car and remote hospital site was successfully established through the 5G network, facilitating sending/receiving data and measurements from both applications (ultrasound unit and video streaming). Core slicing was implemented for a better user experience. Clinical evaluation of the telemedical transmission and applicability of the ultrasound probe was consistently positive.

## Introduction

Digitalization affects almost every aspect of modern daily life including a constantly growing number of health care services along with telemedicine applications. Telemedicine, or telehealth in general, describes the distribution of health-related services and information via electronic information and telecommunication technologies [1]. It allows transmission of medical data or diagnostic information from the patient or a health care provider without the need for the patient and, for example, a medical expert to be present together in the same physical location [2].

With its beginnings in the 1980s, the driving forces of telemedicine have been space travel, expeditions, and military operations. Since then, telemedicine applications have been applied to many different clinical disciplines with three levels to be distinguished: teleconsultation, telepresence, and telemanipulation (telesurgery) [3].

Teleconsultation refers to a video- or audio-based consultation in which the consulting physician represents only a passive (advising) role in the treatment management. In telepresence, the practitioner (eg, a medical expert) actively intervenes in the treatment of the patient over a spatial distance. This can be done, for example, by controlling a diagnostic or therapeutic tool that is used on site during the intervention. The third pillar of telemedicine, telemanipulation (telesurgery), is a further development of telepresence, whereby the active performance of the operation is carried out entirely via a mechatronic system (robot-guided surgery). Teleconsultation thus becomes teleoperation.

Emergency health care is a newer but applicable field for telemedicine [4]. Preclinical point-of-care (POC) information (eg, from an injured patient can be sent from the ambulance car to a remote medical expert in a hospital in real time (and vice versa), regardless of the actual location of the patient prior to arrival [5]. This bidirectional data transmission may consist of physiological data, as well as voice, audio, and video data [6]. Thanks to the constant developments and improvements of broadband wireless communication technologies in recent years, telemedicine applications are nowadays expanding their use range from traditional desktop systems to wireless mobile solutions and thus are a part of smart health care services.

In smart health care systems, Bluetooth, ZigBee, and Wi-Fi are the most noticeable short-range wireless technologies, whereas body area networks, LTE, and WiMAX are long-range technologies used for wireless data transmission [7], enabling various Internet of Things (IoT) applications. For broad IoT adoption, fifth-generation (5G) mobile communication technologies are going to play a major role [8] with significant challenges in terms of interoperability, performance, and security needing to be considered [9].

**Figure 1:**
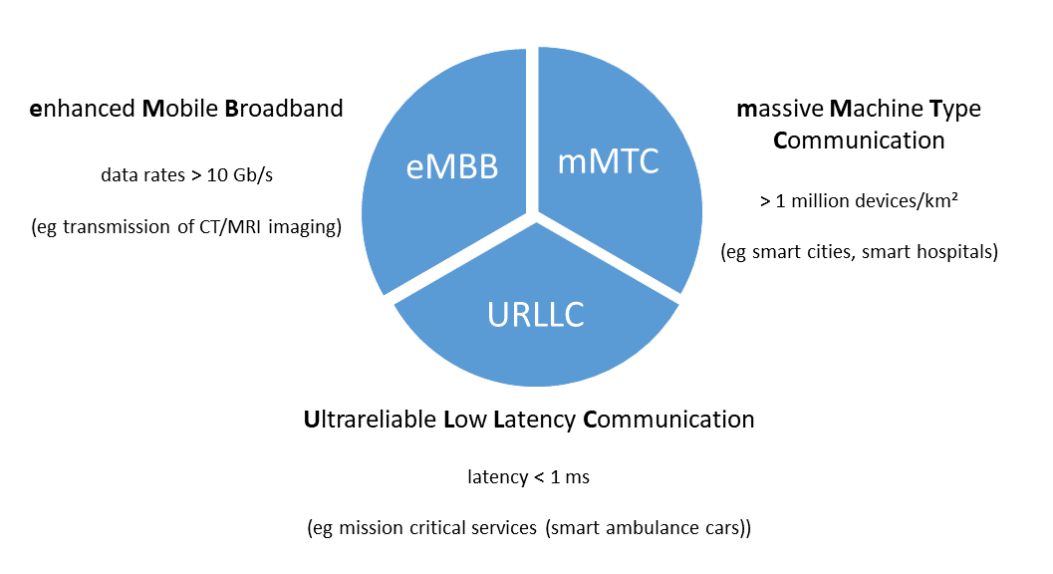
Specifications of 5G new radio providing a set of specifications for the 5G core network as defined in 3GPP Release 15 [11]. CT: computed tomography; MRI: magnetic resonance imaging.

Compared to the current mobile transmission standard 4G/LTE, 5G facilitates a 100 times higher data transmission rate (up to 10 Gbit/s), an extremely low latency (1 ms), and a 1000 times higher capacity [10]. The following types of scenarios (Figure 1) [11] for 5G networks can be classified [12]: enhanced mobile broadband (eMBB), massive machine-type communications, ultrareliable low-latency communications (URLLCs), and wireless regional area networks.

As medical data is considered highly sensitive and requires high security levels, (very) low latency, stable, and ultrareliable data transmission rates, telemedicine applications are linked with strict requirements [12,13]. Since 5G networks include the eMBB and URLLC application profiles, these networks have the potential to fill the gap in mobile (smart) health care [14].

Medical diagnostic ultrasound is a widespread and (compared to other image modalities such as magnetic resonance imaging or computed tomography) inexpensive imaging modality that has become commercially available in portable and mobile configurations in recent years [15]. Thus, mobile ultrasound is the imaging modality best suited for emergency telemedicine applications. In recent years, different mobile ultrasound systems have been developed (eg, for the paramedic care of patients with trauma), allowing the performance of required POC emergency diagnostics prior to hospital arrival [16].

Establishing a wireless connection between an ambulance car (emergency site: paramedics, inexperienced doctors) and a hospital (remote site: medical experts) enables the following medical achievements: increased patient safety due to supervised medical care, improved patient allocation or avoidance of treatment delays by patients’ data analysis and diagnostics prior to arrival, and reduction of (unnecessary) hospitalization rates through preclinical identification of injury severity.

The 5G network has the potential to meet these demands; however, appropriate connectivity setups are needed that take into account optimized bidirectional audio-video streaming according to different parameters such as acceptable image quality and resolution, bandwidth, network slicing, and latency constraints.

**Figure 2:**
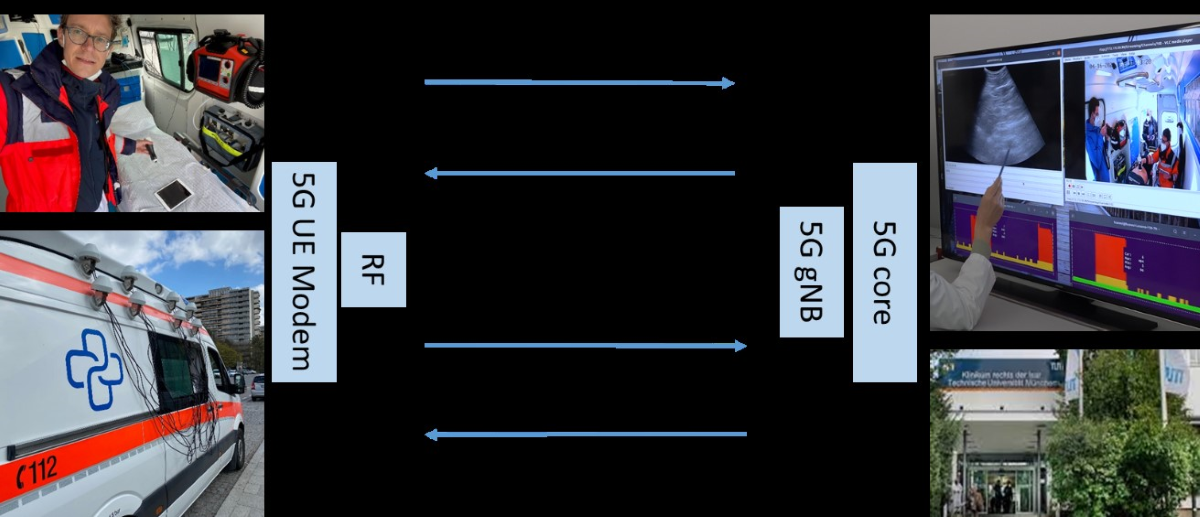
Framework for the 5G field test. Ambulance car (left) equipped with mobile ultrasound and pan, tilt, zoom camera connected to a UE modem. The latter is connected to an RF unit, which is connected to UE antennas on the top roof of the ambulance car. The remote hospital site (right) is connected to the 5G core, which is connected to the gNB. gNB: gNodeB; RF: radio frequency; UE: user equipment.

In this study, we demonstrate a real field test of a 5G framework enabling preclinical diagnostics with mobile ultrasound for emergency patients using 5G network slicing technology. A bidirectional audio-video data transmission between the ambulance car and the hospital was established, combining both 5G-radio and -core network parts to render an end-to-end (E2E) test (Figure 2). Besides technical performance evaluations (key performance indicators [KPIs]) of the 5G network, the medical assessment of transferred ultrasound image quality and transmission latency was examined.

## Methods

### Ethics Approval

This study was conducted in line with the principles of the Declaration of Helsinki. The experimental protocols of the study were approved by the ethics committee (Institutional Review Board) of the Technical University Munich (file AZ 8/16S) and met the guidelines of our responsible governmental agency [17]. All human subjects gave their written informed consent before participating in the study.

### Statistics

Statistical analysis was performed using Excel 2016 XLSTAT (Microsoft Corporation). Descriptive analyses were obtained when applicable. Mann-Whitney *U* tests were calculated for nonparametric distribution. Statistical significance was determined by *P*<.05.

Prior to the performance of the 5G field test, a clinical evaluation of the mobile ultrasound probe (Clarius C3 HD [2-6 MHz], Clarius Mobile Health Corp, Vancouver, BC) was conducted.

### Clinical Evaluation of Mobile Ultrasound Probe

A panel of 20 medical doctors recruited from the departments of surgery and radiology at Klinikum Rechts der Isar, TU Munich experienced in abdominal sonography took part in the clinical evaluation.

They were provided with a questionnaire complied by the authors consisting of 17 topic-specific theses/questions assessing the ultrasound probe in relation to its application feature “telemedicine” (n=6 theses/questions), technical features (n=6 theses/questions), and suitability for clinical use (n=4 theses/questions).

To achieve the highest possible face and content validity of the survey, satisfactory scores of these main three clinical aspects were assessed through a specific 6-point Likert scale, ranging from 1 (very good/strongly agree) to 6 (insufficient/strongly disagree), which has been used previously in similar studies [18].

Performance of at least 5 objective structured clinical examinations with the ultrasound probe according to the Focused Assessment with Sonography for Trauma (FAST) criteria were required per examiner.

### The 5G Field Test

The field test was conducted in Munich, Germany and consisted of a 2-part setup located at the Huawei Munich research center facility: the ambulance car and the remote hospital site. Both were connected through the 5G network using the radio access network (RAN) part of an experimental 5G user equipment (UE) modem and the gNodeB (gNB). The RAN part connects to the core network part located in a cloud data center at the remote hospital site. In the core architecture, a set of network functions, specifically session management functions, user plane functions, and network slicing management functions, whose main features are aligned with the current 3GPP standards, were implemented. The ambulance car was parked in a position of line of sight with the gNB antenna with small buildings located in between as well as cars and pedestrians passing by during the test.

The main target of the field test (Figure 2) was to provide a 5G-based data transfer facilitating bidirectional data transmission for the ultrasound and video source between (1) the ambulance car, equipped with the 5G experimental UE, high-resolution camera (pan, tilt, zoom camera, Hikvision DS-2DE2A404IW-DE3/W, Hangzhou, China) and the ultrasound system, and (2) the remote hospital site (medical expert), equipped with display monitor, camera, and laptop computer.

The field test scenario consists of the parallel transmission of two different applications (ultrasound unit and camera video streaming) with low latency through the 5G network. The carrier frequency was 3.41 GHz, the bandwidth was 40 Hz, and the Modulation Coding Scheme was set to Quadrature Phase Shift Keying. Transmission Time Interval length was 0.5 milliseconds, the intercarrier spacing was 30 KHz, and the antenna gain was 5 dB for UE and 15.5 dB for gNB.

In addition, a traffic saturation to both data channels was simulated by injecting iPerf data with higher throughput. Methods of network slicing were introduced to prove the benefit to network functionality and quality of service (QOS) by prioritizing data traffic and preserving low latency and high-reliability features.

### Clinical Evaluation of the Bidirectional Data Transmission

To assess the medical application of the network KPIs, we performed a qualitative clinical evaluation of the data transmission parameters image quality and latency between ambulance and hospital.

Therefore, the same panel of 20 medical doctors was provided with a structured evaluation form complied by the authors to assess both image quality and latency of the mobile ultrasound, remote ultrasound, and video stream subject to data traffic with/without uplink traffic and use of slicing technology by a specific 6-point Likert scale, ranging from 1 (very good/strongly agree) to 6 (insufficient/strongly disagree). Therefore, the transmitted ultrasound image was recorded (mpeg-4 file) during the field test and made available to the rating participants as was the video recording from the ambulance.

## Results

### Clinical Evaluation of Mobile Ultrasound Probe

A total of 20 medical doctors experienced in ultrasound examinations participated in the clinical evaluation study of the ultrasound probe, which was carried out prior to the 5G field test. All questionnaires were returned for analysis. The application feature “telemedicine” (6 thesis/questions) was generally rated “good”; the aspect of time saving was rated “very good.” Assessment of the ultrasound probe (6 thesis/questions) revealed the overall rating “good”; usability and transmission stability (between ultrasound probe and handheld device; eg, tablet or smartphone by Wi-Fi) were considered to be “very good.” Participants evaluated the clinical application properties (4 thesis/questions) to be “very good” to “good”; in 25% (5/15) of ultrasound examinations a FAST recheck with a second (conventional ultrasound system) was necessary. Table 1 gives a detailed overview of the evaluation results.

**Table 1:** Clinical evaluation of the mobile ultrasound probe with its telemedical and clinical application properties: survey results (N=20 participants).

Application properties		Scores^a^, mean (SD)
**Application feature “telemedicine”**		
	...is helpful	1.9 (0.6)
	...is user-friendly	2.0 (0.7)
	...saves time	1.4 (0.5)
	...provides feedback opportunity	2.3 (0.8)
	...conveys confidence	1.5 (0.5)
	...increases examination quality	1.7 (0.7)
**Technical features ultrasound probe**		
	Haptics	1.7 (0.8)
	Usability	1.3 (0.5)
	Menu navigation	2.0 (0.8)
	Display size	2.0 (0.6)
	Image quality/resolution	2.7 (0.7)
	Transmission stability	1.3 (0.4)
**Suitability for clinical use**		
	Facilitates diagnostics	1.8 (0.7)
	Increases patient convenience	1.6 (0.7)
	Increases physician convenience	1.5 (0.6)
	“Meets the purpose”	1.4 (0.5)

^a^Satisfactory scores were graded on a 6-point Likert scale: 1 to 1.5 (very good/strongly agree), 1.6 to 2.5 (good/agree), 2.6 to 3.5 (satisfying/neutral), 3.6 to 4.9 (sufficient/disagree), and 5 to 6 (insufficient/strongly disagree).

### The 5G Field Test

Bidirectional data transmission between the ambulance car and the remote hospital site was successfully established through the 5G network, facilitating sending/receiving data and measurements from both applications (ultrasound unit and camera video streaming).

**Figure 3:**
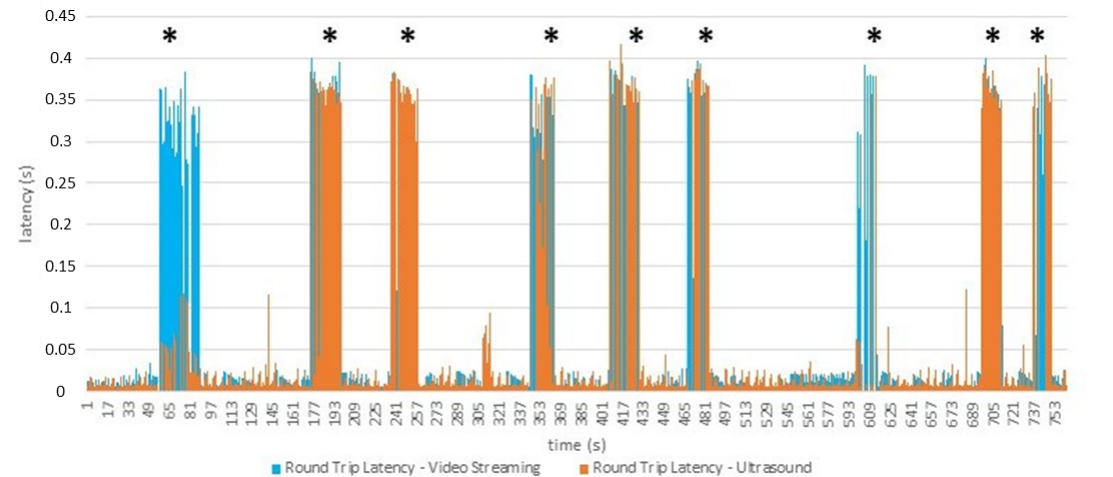
Average end-to-end round trip delay of the two applications: latency and time (seconds). Additional uplink traffic (traffic saturation) marked with *.

The measured average E2E round trip latency, including RAN and core for the data traffic of the two applications is approximately 10 milliseconds (Figure 3). The measured average throughput for the ultrasound image traffic is approximately 4 Mbps and for the video stream 12 Mbps.

**Figure 4:**
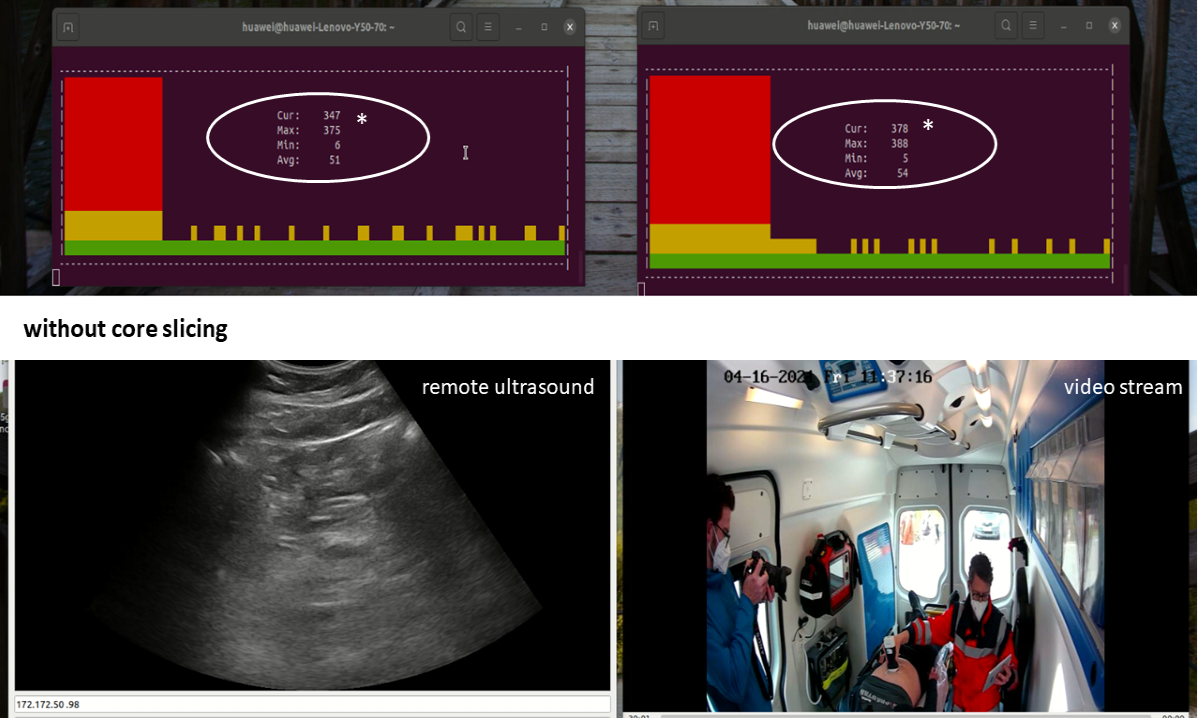
Additional uplink traffic (end-to-end round trip latency *) without core slicing. Ultrasound application (left) and video streaming (right).

Traffic saturation (additional uplink traffic) to compete with both data channels was simulated by injecting iPerf data with higher throughput. For the ultrasound application, a lower video quality and a slower video stream was observed, leading to a heavily interrupted ultrasound image. Additionally, the latency increased up to 400 milliseconds (Figures 3 and 4).

**Figure 5:**
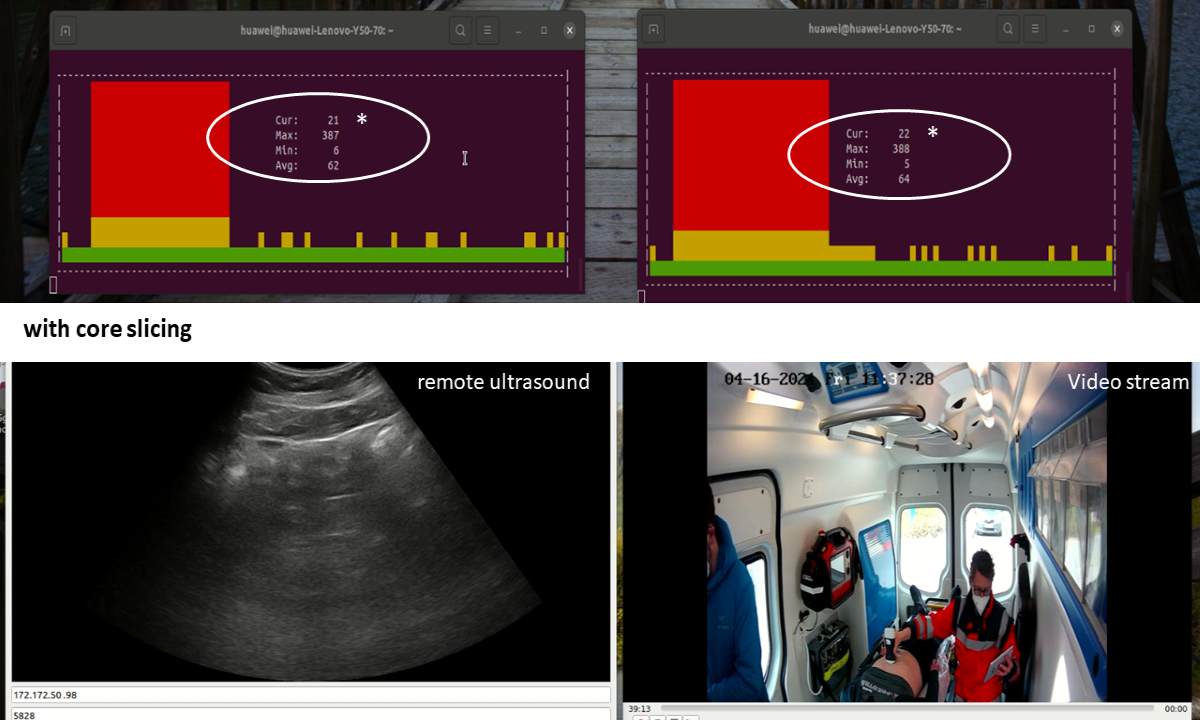
Additional uplink traffic (end-to-end round trip latency *) with core slicing. Ultrasound application (left) and video streaming (right).

Without core slicing, the throughput for the video application was reduced to 8 Mbps after the additional uplink traffic was added. This was also noted for the ultrasound throughput, however, with less influence. By implementing core network slicing, the two applications were prioritized with 2 individual network slices, leading to an immediate quality and latency recovery fulfilling the predefined system requirements (Figure 5).

### Clinical Evaluation of the Bidirectional Data Transmission

Table 2 reveals the survey results of the medical assessment of the bidirectional data transmission characteristics (image quality, latency) between ambulance car and hospital depending on data traffic with/without uplink traffic and use of slicing technology. Whereas the remote ultrasound image quality (hospital site) was not affected by additional uplink traffic, the latency of the displayed image was significantly increased. The same was noted for the video stream transmission. However, using core slicing technology, applicable results for both image quality (*P*=.07) and latency (*P*<.001) were obtained, enabling potentially routine application of the experimental setup. Suitability of image quality and latency for routine clinical application was considered good without uplink traffic (mean 2.2, SD 0.4) and with core slicing (mean 2.2, SD 0.7), respectively.

**Table 2:** Clinical evaluation (N=20 participants) of bidirectional data transmission depending on data traffic with/without uplink traffic and use of slicing technology: survey results.

Data source or characteristics			Uplink traffic							Slicing		
			Without, mean (SD)^a^		With, mean (SD)^a^		*P* value^b^		Score, mean (SD)^a^			*P* value^b^
**Mobile ultrasound (ambulance)**												
	Image quality	1.7 (0.7)		N/A^c^		N/A		N/A			N/A	
**Remote ultrasound (hospital)**												
	Image quality	2.0 (0.7)		2.0 (0.5)		.79		2.2 (0.4)			.28	
	Latency	1.6 (0.7)		3.8 (0.6)		<.001		2.2 (0.6)			<.001	
**Video stream**												
	Image quality	1.9 (0.6)		2.3 (0.4)		.07		2.3 (0.5)			<.001	
	Latency	2.0 (0.4)		3.6 (1.0)		<.001		2.2 (0.4)			<.001	

^a^Satisfactory scores were graded on a 6-point Likert scale: 1 to 1.5 (very good/strongly agree), 1.6 to 2.5 (good/agree), 2.6 to 3.5 (satisfying/neutral), 3.6 to 4.9 (sufficient/disagree), and 5 to 6 (insufficient/strongly disagree).

^b^Mann-Whitney U test.

^c^N/A: not applicable.

## Discussion

### Principal Findings

This usability study of a 5G-enabled emergency setting ensures mobile health care by establishing a bidirectional data transfer (mobile ultrasound imaging, video streaming) between ambulance car (first responder) and remote hospital site (medical expert). Besides technical performance evaluations (KPIs) of the 5G network, a medical assessment of transferred ultrasound image quality and transmission latency was examined. Compared to 4G networks, higher data rates and very low latencies were achieved.

In this study, a real-world 5G field test for a medical application (mobile ultrasound) was performed, examining the benefits of ultralow latency and high reliability (URLLC), which have been proven to be important criteria for providing sensitive and time critical mobile health care applications [12] and slicing. This use case of a 5G-enabled emergency telemedicine application mobile ultrasound consisted of the parallel transmission of 2 different data traffic applications with low latency through a 5G network: first, a mobile ultrasound application providing imaging of the patient with average uplink required service throughput of 5 Mbps, and second, a camera video stream application showing the inside of the ambulance car (patient and doctor) as well as the remote hospital site with average uplink of 10 Mbps and downlink required service throughput of 5 Mbps.

KPIs of the 5G network are consistent to other comparable studies [14]; however, the methods of network slicing that were introduced in our study proves the benefit to network functionality and QOS by prioritizing data traffic and preserving low latency and high-reliability features have not yet been otherwise reported.

Without slicing, the throughput for the video application was reduced to 8 Mbps after the additional uplink traffic was added. This was also noted for the ultrasound throughput, however, with less influence. By implementing network slicing, the two applications were prioritized, leading to an immediate quality and latency recovery and fulfilling the predefined system requirements.

### Comparison With Prior Work

Since its introduction into the industry about 4 years ago, 5G has now begun to show substantial potential and advantages in the health care sector (eg, facilitating intelligent hospital services by real-time patient and asset monitoring [19]). However, there are still certain limitations such as data confidentiality issues, security aspects, and lack of network deployment and support that need to be adequately addressed. For example, from the regulatory point of view, there is currently no bandwidth allocated to emergency services in general [20]. A private band similar to the campus networks in the 3.7 to 3.8 GHz range, which are now available in Germany, would be ideal.

Additionally, 5G technology not only opens new IoT applications for the medical sector [7] but also enhances existing fields such as telemedicine by evolution of cellular networks [21]. Mobile emergency health care apps involving remote assistance by medical experts have stringent connectivity criteria that can be fulfilled by 5G’s promising potential [14]. These include high data rates, massive connectivity, and URLLCs, enabling for example, wireless transmission of mobile ultrasound imaging in real time [2,22] in an emergency. Currently available technologies for data transmission in telemedicine (ethernet/LAN, Wi-Fi, Bluetooth) are limited in terms of transmission rate, latency, and signal stability [10].

To estimate and prove how 5G will restructure the health care system, for example, in the fields of virtual reality and telemedicine [21], representative use cases according to specific, measurable, attainable, relevant, and time-bound objectives [23] are the current worldwide focus of research in this area. An objective of the EU-funded program 5G-VINNI [24] is, for example, boosting the deployment of 5G in Europe by providing E2E facilities, simplifying the vertical industries to pilot use cases and supporting them with evolving infrastructure.

It has been shown that modern telecommunication technologies have the potential to enable the development of sophisticated assistance systems [25] not only for industry 4.0 but also to shape the future of a digitalized health care system. However, to ensure both routine deployment in medical practice and the acceptance of the potential users, systems must meet the prevalent requirements. For instance, we should endeavor to create a noncommercialized band in the range of 3.7 to 3.8 GHz [20].

### Limitations

In this usability study, the 5G system was not exposed by maximum data rates, and the field test was only carried out once without repetition. In addition, clinical evaluation was performed by only 20 participants. Validity and reliability are essential components in the critique of every research and may have an impact on the quantitative and qualitative results of the clinical evaluation.

### Conclusion

Bidirectional data transmission between ambulance car and remote hospital site was successfully established through the 5G network, facilitating sending/receiving data from both applications (ultrasound unit and video streaming). The average E2E round trip latency was 10 milliseconds. The measured average throughput for the ultrasound image traffic was 4 Mbps and for the video stream 12 Mbps. Core slicing facilitated the recovery of quality and latency in case of traffic saturation. The clinical evaluation of the telemedical transmission and applicability of the ultrasound probe was consistently positive (satisfactory score of 1-2 on a 6-point Likert scale; *P*<.001).

## Abbreviations

5G: fifth generation
E2E: end-to-end
eMBB: enhanced mobile broadband
FAST: Focused Assessment with Sonography for Trauma
gNB: gNodeB
IoT: Internet of Things
KPI: key performance indicator
POC: point of care
QOS: quality of service
RAN: radio access network
UE: user equipment
URLLC: ultrareliable low-latency communication
